# Clinical Efficacy and Laboratory Improvement of Bacillus Calmette-Guerin Vaccination on Adult Atopic Asthma *A Cohort Study*

**DOI:** 10.1097/WOX.0b013e31816c8b85

**Published:** 2008-04-15

**Authors:** Eulis A Datau, H Mewengkang, JC Matheos, I Purnawan, M Wibisono, K Wongdjaja, C Wibowo, E Surachmanto, FP Salim

**Affiliations:** 1 Divisions of Allergy-Immunology, Department of Internal Medicine, School of Medicine, Sam Ratulangi University, Manado, North Sulawesi, Indonesia; 2 Divisions of Pulmonology, Department of Internal Medicine, School of Medicine, Sam Ratulangi University, Manado, North Sulawesi, Indonesia

**Keywords:** BCG, symptom/drug score, IFN-γ, IL-4, TGF-β1, adult, atopy, asthma

## Abstract

**Background:**

Recent studies have shown that Bacillus Calmette-Guerin (BCG) vaccination is inversely related to asthma, a Th2 cell-associated with allergic disease, which BCG in humans induces Th1-cell immune responses and prevents airway remodeling.

**Objective:**

To investigate whether thrice BCG vaccinations are clinically effective and could induce laboratory improvement compared with placebo on phase 1 (12 weeks) and single BCG vaccination on phase 2 of the study, then finding out whether the effect might last until 9 months after thrice vaccination and 9 months after single vaccination on adult atopic extrinsic asthma.

**Methods:**

According to the Global Initiative for Asthma criteria, 40 mild to moderate persistent atopic asthma patients were randomly assigned in a double-blind fashion into groups that received intra-dermal injection of 0.1 mL of BCG (n = 20) or 0.1 mL of placebo (n = 20) on the first day. On the first phase, subjects on BCG vaccinations were given intradermal injections 3 times on the deltoid region every 4 weeks. On the second phase, at the 12th week, the placebo group was given BCG vaccination once, and this group became the single BCG group. The symptom score (SS) and drug score (DS), lung function, eosinophil blood count (EBC), total serum immunoglobulin E, interferon-γ (IFN-γ), interleukin 4, and transforming growth factor-β1 (TGF-β1) were examined on the first phase (before the treatment and at the 12th week) and on the second phase (on the sixth and ninth months after the third vaccination for thrice BCG group or after single BCG for control group) to monitor the efficacy.

**Results:**

There were some improvements of asthma SS (*P *< 0.05) and DS (*P *< 0.05), forced expiratory volume in 1 second (*P *< 0.05), peak expiratory flow rate (*P *< 0.05), EBC (*P *> 0.05), IFN-γ (*P *< 0.05), and TGF-β1 (*P *< 0.05) on thrice BCG group compared with prevaccination and with placebo on the first phase and second phase of the study compared with single BCG (formerly placebo).

**Conclusions:**

Based on the previous findings, we could confirm that thrice BCG vaccinations proved to be better than the placebo group and single vaccination. The efficacy of thrice BCG vaccinations on asthma was detected by the improvement of SS, DS, forced expiratory volume in 1 second, peak expiratory flow rate, EBC, IFN-γ, and TGF-β1 until 9 months from the last vaccination without any side effects.

## 

In Indonesia, the prevalence of asthma ranges from 3.8% to 6.9% [1]. In the past decades, treatment of asthma had emphasized long-term suppression of airway inflammation plus relief of symptoms. Inhaled corticosteroids proved to be the most effective agents available on asthma symptomatic control in improving pulmonary function, but potential side effects might occur when increasing the dosage, whereas corticosteroids do not consistently abrogate airway inflammation [2]. Conventional allergen immunotherapy can be effective in many, but not all, patients. Furthermore, an allergist is needed for possible anaphylaxis reaction [3]. Therefore, because of limited treatment of asthma, which is relatively high cost, we need a new modality that is more effective, safer, and cheaper [4].

Bacillus Calmette-Guerin (BCG) is a vaccine used against tuberculosis and as an immunomodulator that primes Th1 lymphocytes to produce cytokines that antagonize atopy in animal models and in human clinical trials [5-9]. Considering atopy as the main risk factor for asthma, we hypothesized that vaccination inducing Th1 responses can be protective against asthma because the Th1 and Th2 cells were reciprocally regulated [10,11]. These hypotheses are based on observations that there was a negative significant correlation between tuberculosis rates and asthma prevalence in children, reduced rating of current asthma symptoms of Japanese school children, [12] and that in murine models of allergen-induced airway eosinophilia, intranasal BCG infection 4 weeks before allergen airway challenges resulted in a 90% to 95% reduction in eosinophilia within the lungs compared with uninfected control mice [10]. There were also a number of reports in Chinese medical literature from the past 30 years that repeated administration of BCG is effective in the management of asthma [13,14]. Also from the regions where tuberculosis is prevalent, [4,15-19] they reported on an association between BCG vaccination and lower rates of atopic disorders. A recent study of Choi and Koh [20] suggested that repeated BCG vaccinations might be more effective in atopic asthma than once. There is no conclusive evidence of the efficacy of BCG against allergy, although the association between BCG and asthma remains a matter of debate [4,20-23].

This article describes an original, randomized, controlled, double-blind, pretest and posttest clinical trial to investigate the efficacy of BCG vaccinations on adult atopic asthma patients during a 12-month cohort study.

## Methods

### Patients

Adult atopic asthma patients were recruited from the Allergy-Immunology outpatient clinic, Prof. RD Kandou Hospital, Sam Ratulangi University, Manado, Indonesia. All patients had asthma for more than 1 year and fulfilled the criteria for mild and moderate asthma set down by the Global Initiative for Asthma 2002 [24]. All patients were reluctant to take fenoterol and budesonide inhaler from the first until the second vaccination, and discontinued budesonide inhaler since the first day of the third vaccination. The subjects were clinically stable for at least 4 weeks before entering this study. Candidates were excluded from the study based on these criteria: chronic obstructive pulmonary disease, acute infectious diseases, smoking at least for 1 year before, malignancies, cell-mediated immune deficiency, human immunodeficiency virus, severe dermatitis on deltoid, severe nutritional deficiency, rheumatoid arthritis, osteoarthritis, systemic lupus erythematosus, multiple sclerosis, previous specific immunotherapy, pregnancy, or lactation. This study was approved by the ethical research committee of the School of Medicine, Sam Ratulangi University. All patients were informed about the experimental procedures and provided written informed consent.

### Study Design

**Figure 1:**
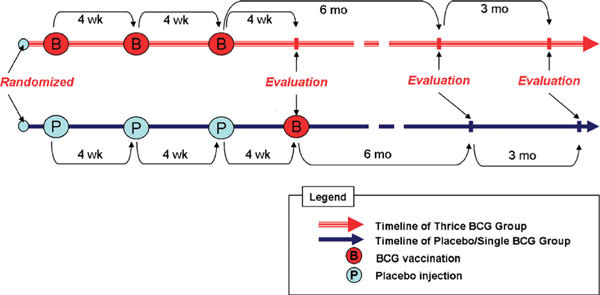
**Trial design**.

This study was designed as a prospective, randomized, placebo-controlled, pretest-posttest, and double-blind clinical trial. The first phase was done from March to August 2004, and the second phase was done from January to April 2005. On the first day before the trial, we carried out spirometry and peak expiratory flow rate (PEFR) determination to diagnose the severity of asthma, and also collection of blood samples for determination of eosinophil blood count (EBC), total serum immunoglobulin E (IgE), interferon-γ (IFN-γ), interleukin 4 (IL-4), and transforming growth factor-β1 (TGF-β1) levels. After the run-in period, the patients were randomly divided into 2 groups (BCG or placebo) and intradermally given BCG or placebo (BCG solvent) vaccination 3 times, each at 4-week intervals. Inevitable development of erythema at the vaccination site in the BCG group posed a problem for the maintenance of the double-blind trial. In regard to this problem, the study coordinator adjusted the asthma medication without examining the vaccination site, and other investigators monitored adverse reactions. Furthermore, all patients were given information to believe that BCG vaccination produced by Biopharma Pharmacy generally produced minimal or no reaction; and none of the patients asked whether they had received BCG vaccination or placebo. The objective and clinical measures were repeatedly done on weekly visits until the end of 12th week and then on the sixth month until the ninth month trial period; EBC and cytokine levels were analyzed again on the 12th week for the first phase, and on the sixth and ninth months for the second phase. Clinical asthma evaluation was derived on 4 symptoms (daytime dyspnea, daytime wheezing, daytime coughing, and nighttime of one of those signs) scoring from 0 to 3, which means 0 for no attack, 1 for mild attack, 2 for moderate attack, and 3 for severe attack. It was called mild attack if asthma did not disturb daily activity, moderate attack if it slightly disturbed daily activities and sleeping, and severe attack if it badly disturbed daily activity and sleeping. Long-acting corticosteroid inhaler (budesonide, 100 μg per puff) and short-acting β2-agonist inhaler (fenoterol hydrobromide, 100 μg per puff) were used. Budesonide was given in 1 puff in the morning for the treatment of mild persistent asthma, whereas for moderate persistent asthma, 1 puff each in the morning and in the evening. Budesonide inhalations were given only until the third vaccination (8 weeks from the first vaccination) to minimize the effect of corticosteroid on immunostimulation. Fenoterol inhalation was given as needed and was used for evaluating drug score (DS) between both groups. Zero for not used, 1 for 1 puff, 2 for 2 puffs, 3 for 3 or more puffs per day. Measurements of PEFR with the mini-Wright peak expiratory flowmeter from Clement Clark International Ltd, England, were done during the 4-week run-in period, 4 weeks after the first, second, and third vaccination, and then every month until 9 months. Measurements of forced expiratory volume in 1 second (FEV_1_) and mid-maximal expiratory volume (MMEV) with spiroanalyzer ST-250 Fukuda Sangyo were done prevaccination and every week until 4 weeks after the third vaccination in the morning at 8:00 to 10:00 am only in the first phase. The highest value among the 3 evaluations was taken as the best one in the study. Atopy diagnosis was based on total serum IgE greater than 120 IU/mL (reference range, < 120 IU/mL for adults 13 years or older). Measurement of IgE was done using an enzyme-linked immunosorbent assay. Measurement of EBC was done with manual techniques using eosin yellow 2%, acetone, and Neubauer counting chamber. Serum IFN-γ, IL-4, and TGF-β1 levels were measured using an enzyme-linked immunosorbent assay (Quantikine K human IFN-γ immunoassay, IL-4 HS 400, HS TGF-β1, R and D systems, Minneapolis, Minn); all were measured on prevaccination, at 12 weeks, at the sixth month, and at the ninth month after the third vaccination. The BCG vaccine contained live attenuated *Mycobacterium bovis *from Pasteur Paris Strain No.1173.P2, produced by PT Biofarma Bandung, Indonesia. The solvent of BCG, saline solution, was used for the placebo vaccination for the first phase. During the study, if there were acute asthma exacerbations, the patients were allowed to have β2-agonist oral/inhalation, aminophylline, with or without oral short-acting corticosteroid. They were also suggested to come every 4 weeks and to avoid allergens in both groups. All local or systemic symptoms including autoimmune responses were noted during the study from 4 weeks, until 12 weeks, and 6 and 9 months after the last vaccination. On the second phase, the patients in the placebo group were each given a single BCG vaccination. The trial design is shown in Figure 1.

### Statistical Analysis

The primary end points showed weekly changes in signs and symptoms and spirometry (PEFR, FEV_1_, and MMEV) above the baseline values, measured at the end of the run-in period. Secondary end points included changes in EBC, IgE, and serum IFN-γ, IL-4, and TGF-β1 levels. The baseline values for each of the variables recorded in the diaries were determined as the averages or sums of weekly values for 4 weeks of the run-in period for the first phase and then every week until 9 months. Categorical variables were compared by using the Fisher exact test for intergroup differences. The differences in continuous variables between the 2 groups were analyzed using the unpaired Student *t *test for parametric data and Mann-Whitney *U *test for nonparametric data. The differences within the same group were analyzed by using the paired Student *t *test for parametric data and the Wilcoxon signed rank test for nonparametric data. A *P *< 0.05 value was considered statistically significant.

## Results

From 52 patients who participated in the run-in period, 40 patients completed the study; 12 patients withdrew because of missing clinical and data visiting. The patient demographics and baseline characteristics were similar for the 2 groups; there are no significant differences in all parameters (Table 1). None of BCG group and placebo group in the first phase reported severe soreness at the site of vaccination. Erythema, manifested as maculopapules (7-10 mm in diameter), occurred in all of the patients in the BCG group, but was not observed in any of placebo group. Neither group showed evidence of severe ulceration, drainage, or lymphadenitis.

**Table 1:** Baseline Characteristics of Prevaccinated Asthma Patients on Both Groups

Characteristics	BCG Group (n = 20)	Placebo Group (n = 20)	*P*
Sex (male/female)	10 (50%)/10 (50%)	8 (40%)/12 (60%)	0.990
Age, yrs (mean ± SD)	34.95 ± 11.55	34.90 ± 12.32	0.675
Body mass index, kg/m^2 ^(mean ± SD)	23.35 ± 3.16	22.97 ± 2.70	0.689
Old healed TBC lesions	0	0	0.5
History of anti-TBC drugs	0	0	0.5
Previous BCG vaccination scar	14 (70%)	15 (75%)	0.517
Asthma triggers, no. (%)			
Dust mite	12 (60)	13 (65)	> 0.05
Cold air	10 (50)	8(40)	> 0.05
Pollutant	9 (45)	8 (40)	> 0.05
Drugs	4 (20)	4 (20)	> 0.05
Seafood	2 (10)	4 (20)	> 0.05
Pollen	1 (5)	0	> 0.05
Duration of asthma, yrs (no. [%])			
1-3	2 (10)	3 (15)	> 0.05
3-5	4 (20)	5 (25)	> 0.05
5-10	9 (45)	8 (40)	> 0.05
> 10	5 (25)	4 (20)	> 0.05
Asthma grading (GINA 2002)			
Mild persistent	6 (30%)	6 (30%)	0.5
Moderate persistent	14 (70%)	14 (70%)	0.5
Hemoglobin, g/dL	14.76 ± 1.53	14.42 ± 1.68	0.508
Eosinophil in differentialcount, %	9.85 ± 6.70	9.80 ± 6.52	0.190

Student *t *test for continuous variables; Fisher exact test for categorical variables.			

**Figure 2:**
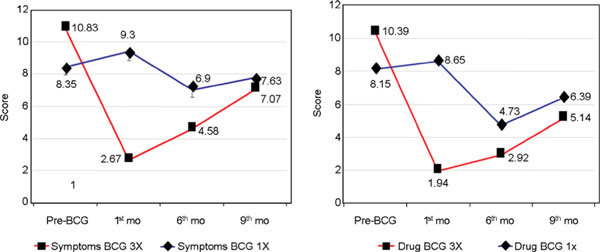
**The SS and DS on BCG vaccination**.

In the thrice BCG group, the weekly symptom score (SS) and DS were significantly decreased beginning on the fourth week until the 12th week as their peaks, and then began to increase on the sixth month until the ninth month, but still better compared with prevaccination (Figure 2). On the contrary, in the single BCG group, SS and DS were slightly increased from the fourth week until the 12th week, and then began to decrease on the sixth month (*P *< 0.05), decreased more on the ninth month, but still better than prevaccination.

**Figure 3:**
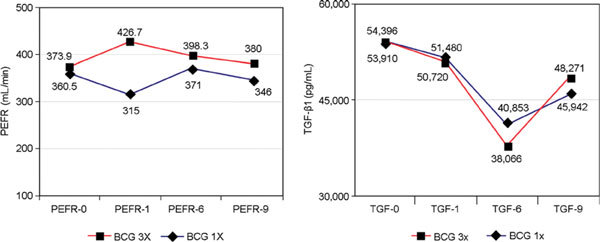
**The PEFR and TGF-β1 levels on BCG vaccination**.

In the thrice BCG group, PEFR was significantly increased from the fourth week until the 12th week (*P *< 0.05), then was slightly decreased from the sixth to the ninth month, but still better than prevaccination. The FEV_1 _and MMEV were all significantly increased until the 12th week prevaccination. On the contrary, in the single BCG group, PEFR was slightly decreased on the fourth week, then was more significantly decreased on the eighth week and the 12th week (*P *< 0.05), but increased again even better than prevaccination on the sixth month, and then decreased again on the ninth month, which was worse than prevaccination; FEV_1 _and MMEV were all significantly decreased on the 12th week (Figure 3).

**Figure 4:**
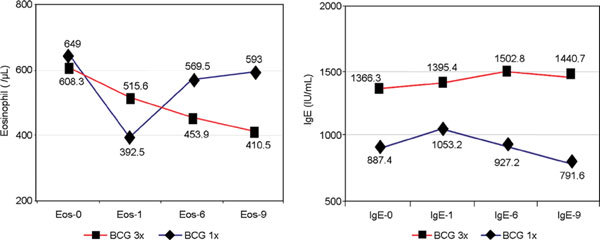
**Blood eosinophil and total IgE levels on BCG vaccination**.

In the thrice BCG group, the EBC was slightly decreased from baseline to 12 weeks, then further decreased on the sixth month, which became statistically significant on the ninth month, but in the single BCG group, the decreases in EBC from the 12th week until the sixth and ninth months were not significant (Figure 4).

In the thrice and single BCG groups, the mean IgE on both phases was slightly increased from the 12th week until the ninth month, except in the single BCG on the ninth month, which was significantly decreased (*P *< 0.05, Table 2, Figure 4).

**Table 2:** Median/Mean Values of Spirometry, Blood Eosinophil Count, TSIgE and Serum Cytokines, Prevaccination and Postvaccination on Both Groups on First and Second Phases

				Postvaccination		
		
Dependent Variables	Prevaccination	4 Wk	8 Wk	12 Wk	6 Mo	9 Mo
Thrice BCG Group						
PEFR (L/min)	360.5 ± 61.1	403.5 ± 71.9*^,†^	409.5 ± 63.9*^,†^	428.0 ± 77.6*^,†^	398.3 ± 101.0*^,‡^	380 ± 86.1^§,‡^
FEV_1 _(L)	2.02 ± 7.6			2.33 ± 0.7*^,†^		
MMEV (L/s)	2.52 ± 1.3			3.12 ± 1.3*^,†^		
Eosinophil count (/μL)	608 ± 493.1			516 ± 522.1^§,‡^	453.9 ± 313.1^§,‡^	410 ± 299.0*^,‡^
TSIgE (IU/mL)	1366 ± 1934.6			1395 ± 1563.7^§,‡^	1502.82 ± 2184.0^§,‡^	1440 ± 2084.4^§,‡^
IFN-γ (pg/mL)	3.55 ± 2.2			4.22 ± 3.8^§,‡^	4.58 ± 2.8*^,†^	4.67 ± 0.5*^,‡^
IL-4 (pg/mL)	0.04 ± 0.039			0.08 ± 0.05*^,‡^	0.09 ± 0.03*^,†^	0.1 ± 0.04*^,†^
TGF-β1 (pg/mL)	54,396 ± 6908			50,720 ± 7111.4*^,‡^	38,066 ± 9279^||,‡^	48,271 ± 9634^||,‡^
Placebo Group					Single BCG Group	
PEFR (L/min)	360 ± 53.3	343 ± 67.4^§,†^	325 ± 63.8*^,†^	315.50 ± 72.9*^,†^	371 ± 73.98^§,‡^	346 ± 95.8^§,‡^
FEV_1 _(L)	2.04 ± 0.65			1.64 ± 0.5*^,†^		
MMEV (L/s)	2.51 ± 1.2			2.01 ± 1.95*^,†^		
Eosinophil count (/μL)	649 ± 580.7			393 ± 358.4*^,‡^	569 ± 553.7^§,‡^	593 ± 582^§,‡^
Total IgE (IU/mL)	887 ± 794.4			1053 ± 863.2^§,‡^	927 ± 1076.3^§,‡^	791 ± 1094.3^§,‡^
IFN-γ (pg/mL)	3.65 ± 1.8			3.85 ± 1.95^§,‡^	5.25 ± 0.90*^,†^	4.81 ± 1.30*^,‡^
IL-4 (pg/mL)	0.08 ± 0.13			0.08 ± 0.11^§,‡^	0.16 ± 0.09*^,†^	0.16 ± 0.13*^,†^
TGF-β1 (pg/mL)	53,910 ± 7525			51,480 ± 6084.9^§,‡^	40,853 ± 12,772^||,‡^	45,942 ± 9453^||,‡^

Values are expressed as mean or median ± SD; *P < 0.05; ^||^*P *< 0.001; ^§^*P *> 0.05.

Bold line separates the first and second phases; ^†^*P *< 0.05 between groups; ^‡^*P *> 0.05 between groups.

**Figure 5:**
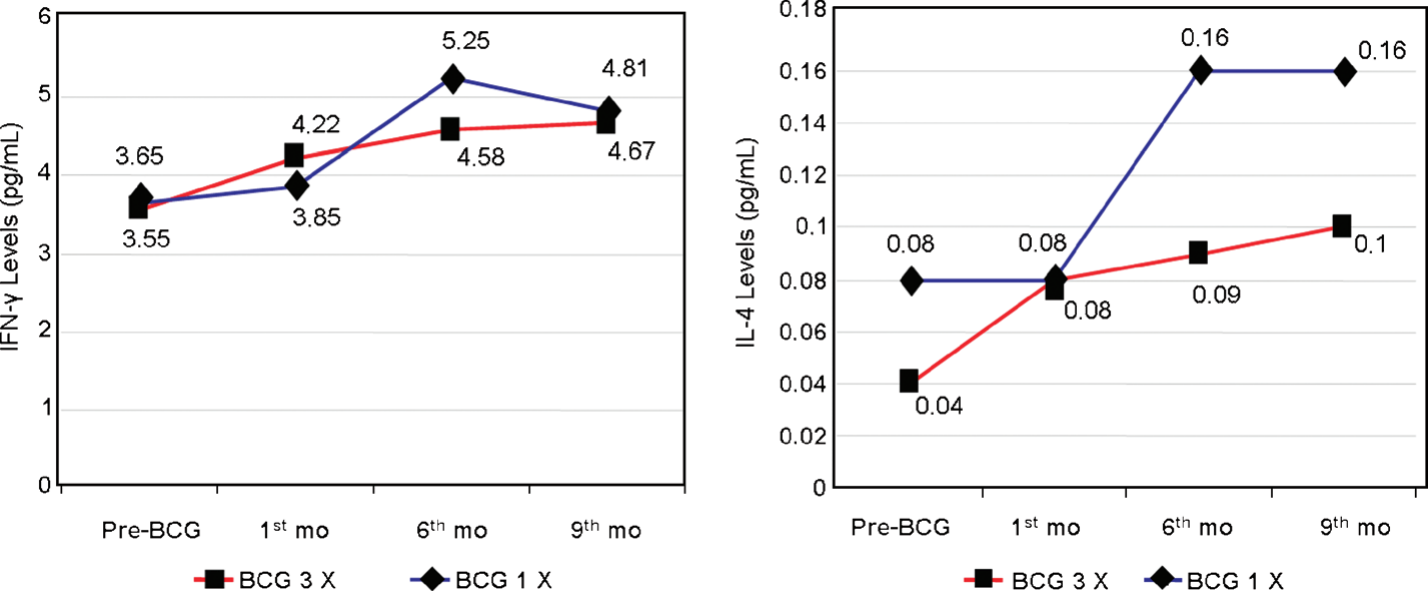
**The IFN-γ and IL-4 levels on BCG vaccination**.

In the thrice and single BCG groups, mean serum IFN-γ was slightly increased on the 12th week but significantly increased (*P *< 0.05) on the sixth month until the ninth month; but between the groups, the differences were not significant except on the sixth month (Figure 5).

Mean serum IL-4 after thrice BCG was significantly increased from the 12th week until the ninth month (*P *< 0.05), except that in the single BCG group on the 12th week, it remained stable and then increased on the sixth and ninth months. Between the groups on the 12th week, there were no significant differences, but on sixth and ninth months, they became statistically significant (Figure 5).

Mean serum TGF-β1 after thrice BCG was significantly decreased beginning on the 12th week; on the sixth and ninth months, they were further very significantly decreased (*P *< 0.001), with its peak on the sixth month. Those patterns were the same as single BCG, but on the 12th week, it was only slightly decreased; between the groups, there were no significant differences until the ninth month (Figure 3).

Besides the scars on the deltoid in the thrice and single BCG groups, which were 7 to 10 mm in diameter, there were no other side effects including autoimmune responses or visiting the emergency department in both groups and in both phases on 9 months' follow-up.

Mean serum TGF-β1 in mild and moderate persistent asthma was not significantly different. The serum IFN-γ correlated negatively with serum TGF-β1 on single BCG vaccination on the sixth month (*r *= -0.63, *P *= 0.001).

## Discussion

Sex ratio between male and female in the BCG group was 1:1, and in placebo, 2:3. Mean age was 34.8 years for both groups; body mass index was normal in both groups. These were similar to reports by Shirtcliffe et al [17] in 2001, where their mean age was 33 years. But Choi and Koh [20] reported a male-female ratio of 4:5 in the BCG group and 1:1 in the placebo group, with a mean age of 47.8 years, subjects were older than those of this study. Body mass index was normal between groups, so there was no negative influence on immune responses, which was similar to the study by Choi and Koh, [4,20] Aaby et al, [16] Shirtcliffe et al, [17] and Marks et al. [19] The allergens on both groups were the same, of which house dust mites were the most prevalent in this study. The duration of asthma in this study was mostly 5 to 10 years, and the clinical diagnosis was mostly moderate persistent asthma (70%) on both groups. In other studies, it was more than 10 years with severe persistent asthma (65%-75%), whereas mild persistent asthma was not included in their studies [19-22].

Choi and Koh [4] reported that weekly SS was significantly decreased only on week 7 in the single BCG group, and there was no difference compared with placebo overall. On the other hand, DS was significantly decreased in the BCG group and in the placebo group. In this study, where the budesonide inhaler was discontinued on both groups, the DS on the placebo group was increased, higher than baseline but not significantly (*P *> 0.05). So SS and DS were significantly decreased in the thrice BCG group starting on week 10 until week 12 (*P *< 0.05) (Figure 2). The influence of discontinuing corticosteroid inhalation in both groups on the eighth week, when the third vaccination was already done, could not be excluded in the placebo group. Spirometry was significantly increased in the thrice BCG group (*P *< 0.05) in this study, this showed a better result than that of Choi and Koh [4] with single BCG.

In the thrice BCG group, the IgE between prevaccination and postvaccination slightly increased (*P *< 0.05); and between postvaccination in both groups was not significantly different, it means that BCG vaccinations could not suppress the IgE synthesis directly. This was similar to the study of Lichtenstein et al (cited in Rale and Patterson [25]) on IgE in pollen immunotherapy, which increased for several months and remained high until 1 to 2 years.

Choi and Koh [20] gave revaccination to the BCG group and for the first time in the placebo group; examination of serum IFN-γ and IL-4 was done before and after 12 weeks from vaccination. In our study, after thrice BCG vaccination, there was slightly increased serum IFN-γ after 12 weeks (*P *> 0.05) and then was significantly increased on the sixth and ninth months (*P *< 0.05) (Figure 5); the increase in serum IFN-γ at 12 weeks in the thrice BCG group was not significantly different from prevaccination in the first phase. Perhaps the 4 weeks' time after the last vaccination was not yet enough to find a significant result compared with the evaluation of Choi and Koh [20] after 16 weeks and may be because their dosage was higher. The significant increase in serum IFN-γ after thrice and single BCG vaccination on the sixth and ninth months reflected the immune responses mediated by Th1 cells. Serum TGF-β1 was significantly decreased (*P *< 0.001) via immune response stimulation of Th1 cells after thrice and single BCG vaccination, which increased the IFN-γ. The decrease of EBC in the thrice BCG group on the 12th week until the sixth month of this study had not occurred significantly, perhaps because of overstimulation by the antigen of thrice BCG. This was similar to the mean IgE, which was still increasing until the ninth month, although in the single BCG group on the ninth month, it began to decrease. On the contrary, during the first phase in placebo group, the EBC was significantly decreased, perhaps influenced by corticosteroid inhalation without any antigen stimulation; and on the sixth and ninth months after single BCG vaccination, the EBC was increased again. These features were similar to the mean IgE under pollen immunotherapy reported by Lichtenstein et al (cited by Rale and Patterson [25]). The mean levels of serum TGF-β1 were also not significantly different between mild and moderate persistent asthma, it means that the increase in TGF-β1 expression has already occurred and began in mild persistent asthma. Serum TGF-β1 has a tendency to stimulate the expression and accumulation of fibro-blast cells, deposition of extracellular matrix and collagen on peribronchial tissues, and the proliferation and hypertrophy of bronchial smooth muscle, and all of these will stimulate hyperreactivity and bronchial obstruction [23]. From the results of the mean PEFR and serum TGF-β1 on the sixth month, we concluded that the peak of improvement of asthma had occurred on the sixth month postvaccination, and after that, the efficacy of BCG vaccination started to decrease in both groups. Choi and Koh [20] reported that the efficacy on PEFR of BCG revaccination after 1 year was better than single vaccination. From our study, we found that BCG has the ability to modulate the asthma immune responses, especially on increasing IFN-γ and inhibiting the expression of EBC, and serum TGF-β1. The studies of Choi and Koh, [4,20] Erb et al, [10] Strannegard et al, [15] and Hoft et al [26] showed the modulating ability of BCG vaccination to influence the immune responses and change the balance of Th1 and Th2. In another study, Hopfenspirger and Agrawal [18] reported on the effect of BCG vaccination on asthma, they did not reveal differences in mean IgE between treated and control groups. Hoft et al, [26] in their study on the effect of single BCG on a normal person, reported the significant increase in serum IFN-γ and the significant decrease in serum IL-4, but they did not find any differences in IgE between the BCG and placebo groups. They proposed that BCG vaccination influences only the immune response that is mediated by Th1 cells, and not by Th2 cells. Cohn et al [27] and Smart et al [28] reported the same thing. This is similar to our finding that BCG vaccinations can improve clinical symptoms and lung function, and significantly increase serum IFN-γ levels, although the mean IgE and serum IL-4 also increased until 6 months. In our study, BCG vaccinations were able to increase serum IFN-γ and suppress serum TGF-β1 and EBC. This was similar to the reports of Choi and Koh [4,20] in Korea, Strannegard et al [15] in Puerto Rico, Aaby et al [16] in Guinea-Bissau, Shirtcliffe et al [17] in New Zealand, Marks et al [19] in Australia, and Zuany-Amorin et al [29] in Brazil. They were all successful in showing immune response modulation to balance the Th1/Th2 in asthmatic patients with BCG vaccine or *Mycobacterium vaccae*. Most of their study subjects (65%-85%) have never had BCG vaccination in childhood. Moehrenschlager et al [30] from the MIRIAM study in Germany also found no hint that BCG vaccination or whole-cell pertussis vaccination may lead to higher prevalences of asthma, allergic rhinitis, eczema, or allergic sensitization at preschool age. Recently, Martignon et al [31] reported a significantly reduced risk of having atopic diseases in adolescents being vaccinated (BCG, diphtheria-tetanus-poliomyelitis, pertussis) in contrast to nonvaccinated controls, and this effect was strongest for atopic eczema and stronger in boys than in girls. On the contrary, the study conducted in regions where BCG vaccination was not given routinely in childhood, as the study of Alm et al [21] in Sweden and Anderson et al [22] in Belgium, could not show the effectivity of BCG on suppressing Th2 cytokines. The increase in serum IFN-γ correlated negatively to serum TGF-β1. The TGF-β1 is a major cytokine of T regulator cells, in particular, it is a potent regulator of fibroblast and myofibroblast function and controls the production of several extracellular matrix protein, including collagens, proteoglycans, and tenascin. Other cell types involved in allergic inflammation as potential sources of TGF-β1 include eosinophils, macrophages, mast cells, neutrophils, endothelial cells, and smooth muscle cells and fibroblasts themselves. The thickening of subepithelial lamina reticularis in bronchial asthma has been related to an increase of fibroblasts in correlation with TGF-β1 expression. Therapeutic treatment of mice with anti-TGF-β1 antibody significantly reduced peribronchial extracellular matrix deposition, airway smooth muscle cell proliferation, and mucus production in the lung (Duvemelle et al, Hashino et al, and Mc Milan et al, as cited by Akdis et al [32]). So the decrease of serum TGF-β1 in this study might contribute to the improvement of clinical signs and symptoms, and lung function, and the decrease in eosinophils. The improvement of clinical signs and symptoms including PEFR could be taken as the best parameter to monitor the efficacy of BCG vaccination. As a conclusion, we could see that thrice BCG vaccination is better than a single one based on lung function from the fourth week until the 12th week, although on the sixth and ninth months, the differences were not significant. Furthermore, on thrice BCG, serum IFN-γ was significantly higher on the sixth month than on single BCG. Then, on the ninth month, the PEFR and serum TGF-β1 began to worsen again, although it was still better than at prevaccination. Maybe after the ninth month for thrice BCG, one could suggest a first booster vaccination.

## Conclusions

The efficacy of thrice BCG vaccination on asthma was demonstrated by the improvements of SS, DS, FEV_1_, PEFR, EBC, IFN-γ, and TGF-β1 until 9 months after the last vaccination, without any side effects.
